# Intensive home treatment in comparison with care as usual: Cost-utility analysis from a pre-randomized controlled trial in the netherlands

**DOI:** 10.1192/j.eurpsy.2021.334

**Published:** 2021-08-13

**Authors:** A. Barakat, J. Cornelis, M. Blankers, A. Beekman, J. Dekker

**Affiliations:** 1 Research, Arkin, Amsterdam, Netherlands; 2 Department Of Psychiatry, Amsterdam UMC, Locatie VUmc, Amsterdam, Netherlands

**Keywords:** economic evaluation, randomized controlled trial, intensive home treatment, emergency psychiatry

## Abstract

**Introduction:**

The implementation of Intensive Home Treatment (IHT) aims to decrease the pressure on acute inpatient services that could lead to prevent hospitalization and reduce the number of hospitalization days and, ultimately, reduce cost in the mental health services. Although there are studies assessing the effectiveness of IHT, there is a shortage of research studying the cost-effectiveness.

**Objectives:**

The aim of this study is to present an cost-utility analysis of IHT compared to care as usual (CAU)

**Methods:**

Patients between 18 and 65 years of age whose mental health professionals considered hospitalization were included. These patients were pre-randomized in either IHT or CAU and followed up for 12-months. For this study, the base case analysis was performed from the societal and healthcare perspective. For the cost-utility analyses the Euroqol 5D was used to calculate quality adjusted life years (QALYs) as a generic measure of health gains.

**Results:**

Data of 198 patients were used. From a sociatal perspective, the cost-utility analysis resulted in an incremental cost-effectiveness ratios (ICERs) of €58 730, and a 37% likelihood that IHT leads to higher QALYs at lower costs. The probability of IHT being cost-effective was >50% if there was no willingness to pay more for extra QALY than in the current situation under CAU.

**Conclusions:**

Professionals working in crisis care are able to offer IHT with the same effect as other crisis care interventions at lower costs. IHT seem to be cost-effective compared with CAU over 52 weeks follow-up for patients who experience psychiatric crises.

**Disclosure:**

No significant relationships.

